# Facebook Intrusion as a Mediator Between Positive Capital and General Distress: A Cross-Cultural Study

**DOI:** 10.3389/fpsyt.2021.667536

**Published:** 2021-06-17

**Authors:** Aneta Przepiórka, Agata Błachnio, Mark Sullman, Oleg Gorbaniuk, Nicolson Yat-Fan Siu, Tetiana Hill, Maria-Eugenia Gras, Antonios Kagialis, Yanina Lisun, Maité Díaz-Peñaloza, Denisse Manrique-Millones, Militsa Nikiforou, Galina S. Evtina, Joanne E. Taylor, Burcu Tekes, Laura Šeibokaite, Lisa Wundersitz, Fran Calvo, Sílvia Font-Mayolas

**Affiliations:** ^1^Institute of Psychology, The John Paul II Catholic University of Lublin, Lublin, Poland; ^2^School of Humanities and Social Sciences, University of Nicosia, Nicosia, Cyprus; ^3^Faculty of Psychology, University of Economics and Human Sciences in Warsaw, Warsaw, Poland; ^4^Department of Counselling and Psychology, Hong Kong Shue Yan University, Hong Kong, China; ^5^Hertfordshire Business School, University of Hertfordshire, Hatfield, United Kingdom; ^6^Department of Psychology, Quality of Life Research Institute, Universitat de Girona, Girona, Spain; ^7^Department of Journalism and Advertising, Kyiv National University of Trade and Economics, Kyiv, Ukraine; ^8^Instituto de Investigación de Psicología - Universidad de San Martín de Porres, Lima, Peru; ^9^Grupo de Investigación en Comunicación y Salud, Instituto de Investigación Científica, Universidad de Lima, Lima, Peru; ^10^School of Sciences, University of Central Lancashire, Larnaca, Cyprus; ^11^Industrial University of Tyumen, Tyumen, Russia; ^12^School of Psychology, Massey University, Palmerston North, New Zealand; ^13^Department of Psychology, Başkent University, Ankara, Turkey; ^14^Department of Psychology, Vytautas Magnus University, Kaunas, Lithuania; ^15^Centre for Automotive Safety Research, The University of Adelaide, Adelaide, SA, Australia; ^16^Department of Pedagogy, Quality of Life Research Institute, Universitat de Girona, Girona, Spain

**Keywords:** Facebook intrusion, positive capital, self-esteem, self-control, ego resiliency, general distress, cross-country study

## Abstract

**Background:** Social networking sites (SNSs) play an important role in many aspects of life nowadays, and it seems to be crucial to explore their impact on human well-being and functioning. The main aim of the study was to examine the mediating role of Facebook intrusion between positive capital and general distress. Positive capital was considered as comprising self-esteem, ego-resiliency, and self-control, while general distress was seen as having three dimensions: depression, anxiety, and stress.

**Methods:** The sample consisted of *N* = 4,495 participants (*M* = 22.96 years, *SD* = 5.46) from 14 countries: Australia, Cyprus, Greece, Hong Kong, Lithuania, New Zealand, Peru, Poland, Russia, Spain, Turkey, Ukraine, United Kingdom, and United States. We used the following methods: the Facebook Intrusion Questionnaire (FIQ), the Self-Esteem Scale (SES), the Brief Self-Control Scale (SCS), The Ego Resiliency Revised Scale and the Depression, Anxiety, and Stress Scale-21 (DASS-21).

**Results:** We found that Facebook intrusion was a mediator between self-esteem and general distress and between self-control and general distress.

**Limitations:** The present study was based on a cross-sectional study, and the measures used were self-report measures. The majority of the participants were recruited using convenience sampling.

**Conclusions:** The present findings contribute to a better understanding on how the social media have impact on individual mental health. Implications for future studies are discussed.

## Introduction

Social networking sites (SNSs) play an important role in modern society by providing a tool for communication, education, and entertainment in professional and private life (1). Facebook is one of the most popular and widely used SNSs. Since its launch in 2004, it has profoundly changed the way people share information, interact with each other, and spend their time. A growing body of research clearly highlights its profound impact on human social, physical, and emotional functioning (2). There are studies that support the notion of Facebook's positive effect on social capital and psychological well-being (3, 4). It has been found that social support on Facebook has a positive impact on satisfaction with life (5). However, there are also a number of studies that reveal the dark side of its use including addictive power (6–8). A recent meta-analysis showed a positive relationship between SNS use (i.e., variables such as time spent on social networking sites or the frequency of checking) and depression (9).

In our research, we refer to Facebook intrusion, which is defined as excessive Facebook use manifesting itself, among other symptoms, in the loss of control, tolerance, and disruption of everyday routine (10). The definition of this term is based on the criteria used in behavioral addictions, such as withdrawal, relapse and reinstatement, and euphoria (10). Cross-cultural studies indicate that culture should be considered when analyzing and interpreting the findings on Facebook use, although a study by Błachnio et al. (11) outlined some universal personality and cultural patterns of Facebook intrusion (11). At the country level, uniqueness and low context were associated with Facebook intrusion (the former negatively and the latter positively), whereas at the personality level, conscientiousness and emotional stability were linked to Facebook intrusion.

Social media are a relatively new phenomenon, and their impact on human well-being and functioning has not been fully explored. The present study adds to the existing knowledge by posing a question on the possible determinants and impact of Facebook intrusion. The main aim of the present study was to examine the mediating role of Facebook intrusion between positive capital and mental health problems. What we understood by positive capital was certain psychological characteristics—namely, self-esteem (12), ego resiliency (13), and self-control (14)—that can be regarded as the inner strengths that not only contribute to mental health, in general, but might also reduce the negative effect of Facebook use. We operationalized mental health problems as depression, anxiety, and stress, jointly labeled as general distress; this one-factor solution was consistent with other results (15). Self-esteem, ego resiliency, and self-control were chosen because of their significant relationship to Facebook use and mental health conditions [e.g., (16)]. We included gender as a control variable because it is related to new media addiction [e.g., (17, 18)]. There are no unambiguous research results identifying clear differences in the level of addiction between women and men. Some studies suggest that differences stem from what applications individuals of a given sex most often use (19).

### Positive Capital and Mental Health

Personality dimensions and personal resources, including self-esteem, ego resiliency, and self-control, were evaluated as mechanisms promoting individual differences in mental health as reported in other studies [e.g., (20, 21)]. According to Seligman and Csikszentmihalyi (22), the focus in the term “positive capital” is placed on strengths rather than weaknesses, vitality, and mental health.

Self-esteem can be conceptualized as the feeling that one is an object of primary value in a meaningful universe (23). Researchers found that self-esteem was negatively related to depressive symptoms (24) and anxiety (25). Eisenbarth (26) also demonstrated that college students with low self-esteem were more likely to develop depressive symptoms and experience high stress because they did not feel confident about their competence. In contrast, higher self-esteem buffered against depressive symptoms when under high level of stress. Self-esteem also has an anxiety-buffering function. In the experimental study by Greenberg et al. (23), participants high in self-esteem reported lower anxiety in response to threatening images of death and had lower arousal in response to the threat of electric shock (23).

Ego resiliency is the ability to flexibly and resourcefully adapt to internal and external stressors and to quickly recover from stress (13, 27). Individuals with low ego resiliency tend to lack diversity in healthy strategies in coping with life's challenges (28). Researchers found that ego resiliency was negatively related to the level of anxiety and perceived stress (29, 30). Cole et al. (31) reported that ego resiliency was negatively related to anxiety and depression. Ego resiliency also mediated the negative relationship between the levels of social stigma and depressive symptoms in adolescent dropouts (32).

Self-control can be defined as the ability to concentrate, inhibit impulses, and delay gratification (33). Galla and Wood (34) found that self-control positively predicted exposure and reactivity to daily stress and that it negatively predicted adaptive responses to stress. Self-control is an important ability to exert control over unwanted behaviors in the successful pursuit of goals and in daily routine (35). It helps to focus energy on pursuing a goal by reducing the harmful effect of temptations. It enables a person to achieve goals, provides the energy necessary to accomplish daily tasks, and is beneficial in different domains such as academic performance, consumer behavior, or emotional control (36).

### Facebook Intrusion as a Mediator

Based on a review of the pertinent literature, we expected that the positive effects of psychological capital would reduce the negative impact of excessive Facebook use on mental health. A previous study (37) showed that low self-control was related to Facebook intrusion. Low self-esteem was found to be one of the predictors of Facebook intrusion (16, 38, 39). Cudo et al. (40) examined the relationship between impulsivity (as a dimension of self-control) and Facebook addiction. Their results indicated that Facebook addiction was predicted by a higher level of impulsivity, which suggested that individuals with lower self-control were more likely to develop Facebook addiction. Lim (41) found that ego resiliency had a negative impact on SNS addiction tendency. Sindermann et al. (42) reported that individuals who had higher scores in self-discipline had a lower tendency toward Facebook-use disorder. Similarly, Cudo et al. (43) found that problematic Facebook use was positively related to maladaptive schemas, which included insufficient self-discipline and approval seeking.

There is evidence showing that Facebook intrusion has a negative impact on human functioning in terms of physical and mental health (44). Meta-analyses have shown Facebook use to be related to a number of mental health outcomes, including anxiety, depression, disordered eating, and negative body image (45). Researchers found that social anxiety (46) and depression (47, 48) could be caused by Facebook addiction. A meta-analysis study (49) also revealed a positive correlation between problematic Facebook use and psychological distress, which included anxiety and depression. In contrast, an experimental study (50) demonstrated that cognitive and affective well-being were enhanced by quitting Facebook. Participants who did not use Facebook for a week reported higher life satisfaction and improvements in emotional life.

Based on the findings discussed above, we formulated the following hypotheses:

H1: Facebook intrusion mediates between self-esteem and general distress in such a way that self-esteem reduces the level of Facebook intrusion, which translates into a lower level of general distress.
H2: Facebook intrusion mediates between self-control and general distress in such a way that self-control reduces the level of Facebook intrusion, which translates into a lower level of general distress.
H3: Facebook intrusion mediates between ego resiliency and general distress in such a way that ego resiliency reduces the level of Facebook intrusion, which translates into a lower level of general distress.

**Figure 1:**
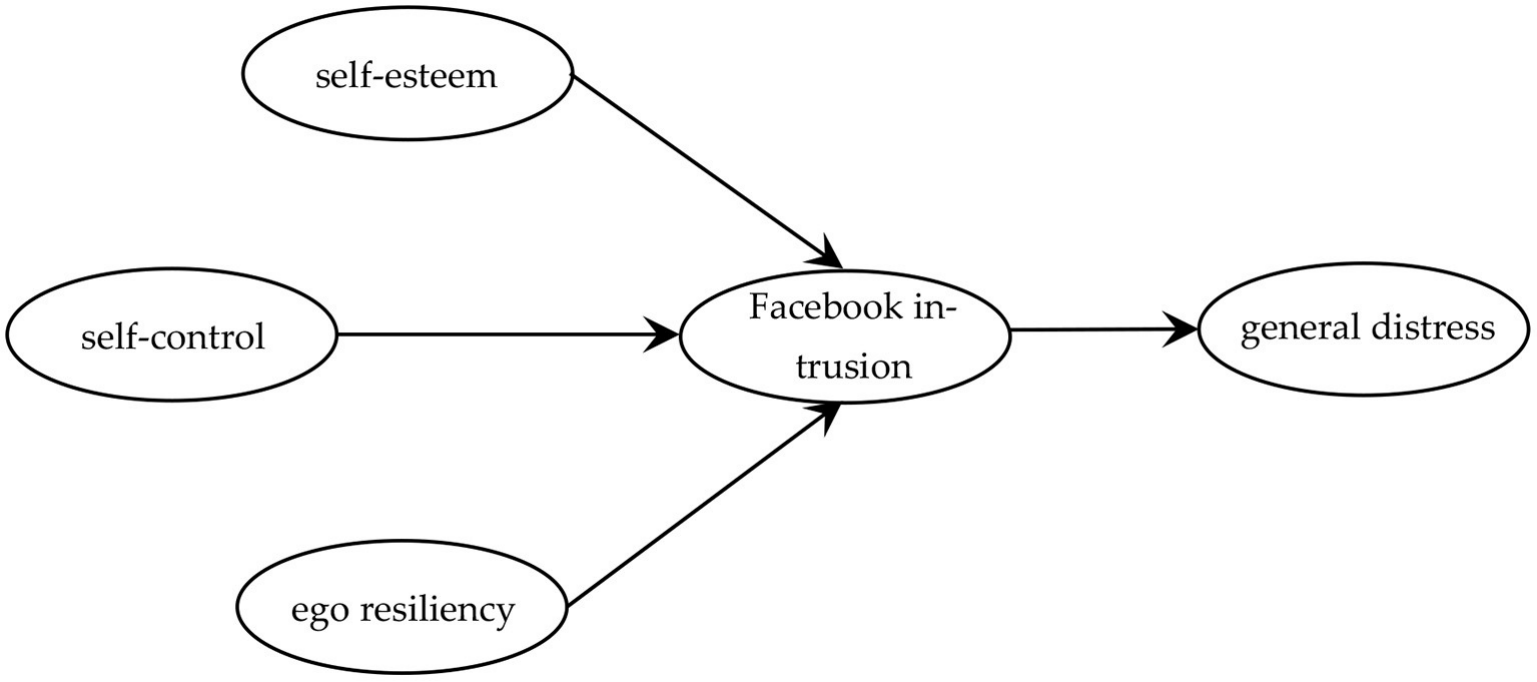
Theoretical associations between the variables.

The associations are presented in Figure 1.

## Materials and Methods

### Participants

A sample of *N* = 4,495 respondents took part in the study; 27.4% of them were men, 72.0% were women, and 0.6% identified themselves differently in terms of gender. Participants' mean age was *M* = 22.96 years (SD = 5.46). Data were collected from 14 countries: Australia, Cyprus, Greece, Hong Kong, Lithuania, New Zealand, Peru, Poland, Russia, Spain, Turkey, United Kingdom, Ukraine, and United States.

### Procedure

The presented results are part of a bigger project that involves the same 14 countries. Its other results not related to the aim of this study have been published elsewhere. The present study was conducted in local languages with a back-translation process (from English into local languages) being used. To reach a large group of respondents, varied in terms of sociodemographic characteristics, we applied snowball sampling. After the electronic version of the questionnaires were prepared, the link to the research site was sent out *via* the Internet. A special invitation to the study was posted on the university's website. Participants volunteered to take part in the study and received no monetary reward. They were informed about the anonymity of the study, and the study was approved by the institutional research ethics board.

### Measures

We used several measures in the study.

To measure Facebook intrusion, we used the Facebook Intrusion Questionnaire (10), which is based on behavioral addiction components and on a scale measuring phone involvement. The scale consists of eight items (e.g., “I have been unable to reduce my Facebook use”), which are rated on a seven-point Likert scale (1 = *completely disagree* to 7 = *completely agree*) measuring the relations between Facebook involvement tendency and eight aspects of behavioral addiction, namely: cognitive salience, behavioral salience, interpersonal conflict, conflict with other activities, euphoria, loss of control, withdrawal, and relapse and reinstatement. The values of Cronbach's α ranged from 0.76 to 0.91, depending on the country.

To measure self-esteem, we used Rosenberg's Self-Esteem Scale (12), which is answered on a four-point Likert scale (1 = *completely agree* to 4 = *completely disagree*). The scale consists of 10 items (e.g., “I feel that I have a number of good qualities”) and yields an overall evaluation of a person's self-esteem. Cronbach's α ranged from 0.83 to 0.91.

The Brief Self-Control Scale (14) was used to measure dispositional self-control, which is defined as the ability to influence one's inner responses and refrain from undesired behavioral tendencies. The scale consists of 10 items (e.g., “I am good at resisting temptation”) and Cronbach's α has ranged from 0.71 to 0.84.

The Ego Resiliency Revised Scale (13) consists of 14 items (e.g., “I am generous with my friends”), which were rated on a seven-point scale, which ranges from 1 = *never* to 7 = *always*. Cronbach's α ranged from 0.75 to 0.85.

The Depression, Anxiety, and Stress Scales-21 (51) has 21 items, which are rated on a four-point Likert-type scale (0 = *did not apply to me at all* to 3 = *applied to me very much or most of the time*). The scale consists of three subscales: depression, anxiety, and stress. Following the solutions presented in other studies (52), we applied a bifactor structure in our analyses so as to get a broader view of the associations investigated and to assess mental health problems in general. Cronbach's α ranged from 0.91 to 0.95.

### Statistical Analyses

We conceptualized the data as a two-level structure in which individual respondents were nested within countries of residence. To test the hypotheses, we used Mplus 7.3 (53). To examine individual-level phenomena, we investigated pure level 1 effects (individual effects) only, without considering level 2 variables and their influence on level 1 variables. Therefore, we used group mean centering to control for the differences between countries: group effects are accounted only by the variance term.

We used the ML estimator in a two-level analysis (54). Since the tested model contained 70 observed variables and eight latent variables and is in fact the sum of five measurement models, we used the following criteria to assess the model's goodness of fit: (1) comparative fit index (CFI) equal or higher than 0.90 (optimally it should be equal to or higher than 0.95), (2) root mean square error of approximation (RMSEA) and standardized root-mean-square residual (SRMR) lower than 0.07 (optimally they should be lower than 0.05) (55, 56).

We performed a confirmatory factor analysis to determine the fit of the measurement model. Next, a full mediation model was tested, followed by a partial mediation model. We used gender as a control variable (i.e., a covariate; 1 = female, 0 = male) in each of the structural models. The support for the less restrictive, partial mediation model will be a significant improvement in the fit of the model to the data in the scaled difference chi-square test. To determine the mediating effects, we broke down model parameters into direct and indirect effects.

## Results

The composite reliability of the measures and correlation between the variables are presented in Table 1. Facebook intrusion was found to be positively related to general distress. It was also negatively correlated with self-esteem, ego resiliency, and self-control.

**Table 1:** Reliability of the scales and within-level correlations between latent variables.

**Variables**	**ω**	**1**	**2**	**3**	**4**
1. Facebook intrusion	0.86	–			
2. Self-esteem	0.88	−0.09^**^	–		
3. Self-control	0.79	−0.28^**^	0.44^**^	–	
4. Ego-resiliency	0.81	−0.05^*^	0.52^**^	0.20^**^	–
5. General distress	0.93	0.19^**^	−0.52^**^	−0.46^**^	−0.22^**^

*ω, composite reliability*.

* *p < 0.01*.

** *p < 0.001*.

**Figure 2:**
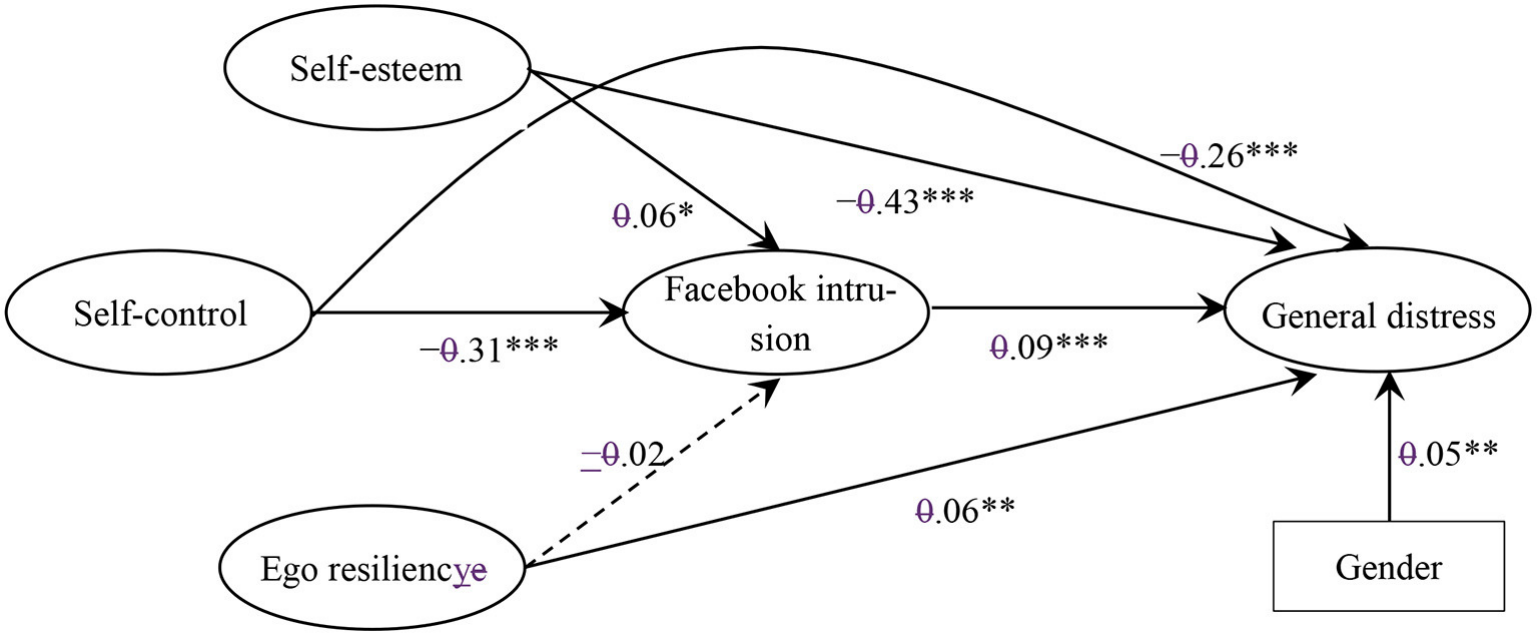
Standardized path coefficients for the within-level structural partially mediated mode l. *p < 0.05. **p < 0.01, ***p < 0.001.

The measurement model showed acceptable fit to the data (see Table 2). Fit indices for the fully and partially mediated structural models are reported in Table 2. They indicate the acceptable fit of both models to the data. The scaled chi-square test of differences between the models is statistically significant (Δχ^2^ = 244.37, Δdf = 3, *p* < 0.001), which argues in favor of the partially mediated model. The standardized path coefficients for the within-level partially mediated model are reported in Figure 2. All paths except the one between ego resiliency and Facebook intrusion are statistically significant. The results support the hypothesis that Facebook intrusion mediates the relationship between self-control and general distress.

**Table 2:** Measurement and structural within-level model fit indices.

	**Overall fit indices**			
**Model**	**χ^2^(*df*)**	**CFI**	**RMSEA**	**SRMR (within)**
Measurement model	11,257.64 (1,710)	0.907	0.036	0.045
Full mediation	11,968.39 (1,776)	0.901	0.036	0.046
Partial mediation	11,724.02 (1,773)	0.904	0.036	0.045

There are indirect effects linking self-esteem (b = 0.005, SE = 0.002, *p* < 0.05) and self-control (b = −0.026, SE = 0.005, *p* < 0.001) with general distress via Facebook intrusion. Facebook intrusion, however, does not mediate the relationship between ego resiliency and general distress (b = −0.002, SE = 0.002, *p* = 0.361). This means that hypotheses H1 and H2 about the mediating role of Facebook intrusion were supported only for self-esteem and self-control, while hypothesis H3 was not supported. Self-esteem, self-control, and ego resiliency explain 8.2% of the variance in Facebook intrusion (R^2^ = 0.082, SE = 0.010, *p* < 0.001). As regard the variance in general distress, they explain 34.6% (R^2^ = 0.346, SE = 0.018, *p* < 0.001).

## Discussion

In our study, we examined the mediating role of Facebook intrusion between positive capital and mental health problems. We operationalized positive capital as comprising self-esteem, ego resiliency, and self-control and considered mental health problems in terms of three dimensions: depression, anxiety, and stress. The study showed that Facebook intrusion was a mediator between self-esteem and mental health problems and between self-control and mental health problems.

We found a significant but small mediating effect of Facebook intrusion between self-esteem and general distress (H1), but the direction was opposite of the expected one. It is possible that Facebook is generally for people to promote themselves and to create their image (39, 57). Cudo (58) found that Facebook intrusion could be negatively predicted by self-esteem, and this relationship was fully mediated by depression. Błachnio et al. (7) suggested that Facebook intrusion was predicted by depression, but the reverse relationship between these factors is also possible. Previous studies have shown that problematic social media use could predict an increase in depressive symptoms (59, 60). A meta-analysis (61) also revealed a complex relationship between online social networking and depression, since these variables can be influenced by other factors. For instance, depression can be caused by negative comparison with others, which was found to increase rumination when using Facebook (62). In addition, several studies have found that frequent use of SNSs could negatively affect mental health by increasing depression, anxiety, psychological distress, and suicidal ideation (63–65). These studies suggested that general distress was predicted by use of SNSs, which is consistent with the findings of our study.

Moreover, we found that Facebook intrusion was a mediator between self-control and general distress (H2). However, Hofmann et al. (66) reported a different role, suggesting that self-control could moderate the effects of media use on well-being. They indicated that habitualized social media use could increase the risk of media-related self-control failure. Most social media users exhibit strong automatic approach reactions, which increase impulsive behavior. The authors further suggested that short-term negative effects, such as stress and frustration, would increase while immediate gratification (i.e., checking Facebook) was delayed by a procrastinated task (i.e., writing a term paper). Nevertheless, previous studies have found that problematic behaviors (67), problematic Internet use (68, 69), and Internet addiction (70) were found in individuals with low self-control. Griffiths (71) further revealed that Internet addiction was not homogenous and that it was caused by low self-control. Although various studies investigated the effect (72, 73) and related factors [e.g., personality; (74)] of Facebook use, Firat (75) points out that the relationship between self-control and Facebook use has seldom been analyzed. Recently, Cudo et al. (40) found self-control was predictive of Facebook intrusion. Błachnio and Przepiorka (37) also established that the risk of Facebook addiction was caused by psychological characteristics. which included insufficient self-control and a low level of failure-related action orientation.

Also, contrary to our expectations (H3), there was no mediating effect of Facebook intrusion between ego resiliency and general distress. The reason for its absence might be that the construct was too broad to account for the mediating effect of Facebook use.

### Limitations

The present study is not free from limitations. First, it was based on a cross-sectional study, which means it cannot yield conclusions about causal effects. Second, the measures used in the study were self-report measures. It is recommended that future studies utilize a longitudinal approach in order to determine which is the cause and which is the outcome. Third, the majority of the participants were recruited using convenience sampling; however, this age group (19–25) constitutes the largest group of Facebook users. In future studies, it would be interesting to distinguish between active and passive use and to determine the differences between them in their relations to mental health and psychological well-being. Last, future studies should include more countries, so as to get a more universal pattern of relationships.

## Conclusions

In conclusion, our study has shown that Facebook intrusion plays an important role in the relationship between positive capital and mental disorder. The mediating role of Facebook intrusion was confirmed in the relationship between self-control and general distress and between self-esteem and general distress. As this was a cross-cultural study involving 14 countries, the model seems to be universal and cross-cultural. These findings can be useful for practitioners and therapists in the process of motivating people to reduce addictive tendencies for social media use. The research results highlight the preventive role of certain personal resources that should be developed in those at risk of developing problematic use of social networking sites. The present research also suggests that future interventions should help those using social networking sites to build a stable self-image and to strengthen their self-control.

## Data Availability Statement

The raw data supporting the conclusions of this article will be made available by the authors, without undue reservation.

## Ethics Statement

The studies involving human participants were reviewed and approved by the Institutional Ethics Committee. The patients/participants provided their written informed consent to participate in this study.

## Author Contributions

AP conceptualized the study, did the project administration, and acquired funding. OG prepared the methodology, software for the study, and formal analysis. AP, AB, and MS performed validation and supervision. AP, AB, MS, NS, TH, M-EG, AK, YL, MD-P, DM-M, MN, GE, JT, BT, LS, LW, FC, and SF-M performed investigation for the study. AP and MS did data curation. AP, AB, OG, and NS prepared and wrote the original draft. AP, AB, MS, NS, TH, M-EG, AK, YL, MD-P, DM-M, MN, GE, JT, BT, LS, LW, FC, and SF-M reviewed and edited the original manuscript. AP, AB, and OG did visualization. All authors have read and agreed to the published version of the manuscript.

## Conflict of Interest

The authors declare that the research was conducted in the absence of any commercial or financial relationships that could be construed as a potential conflict of interest.
